# Anti-inflammatory drug and prebiotics alter metabolic inflammation in fattening ground squirrels and outbred mice

**DOI:** 10.3389/fphys.2026.1802487

**Published:** 2026-07-29

**Authors:** Ashleigh A. Kucharski, Celine E. Brice, Noah A. Hayes, Khrystyne N. Duddleston, Courtney C. Kurtz

**Affiliations:** 1 Biology, University of Wisconsin Oshkosh, Oshkosh, WI, United States; 2 Biological Sciences, University of Alaska Anchorage, Anchorage, AK, United States

**Keywords:** gut, inulin, mesalazine, pectin, WAT

## Abstract

Metabolic inflammation is an inflammation event that occurs during fattening driven by diet-induced changes to the microbiota leading to increased permeability of the gut barrier and infiltration of bacterial products. This triggers inflammation in the gut and distant tissues (i.e., white adipose [WAT]). We have previously shown that 13-lined ground squirrels (*Ictidomys tridecemlineatus*), obligate hibernators, develop metabolic inflammation during their fattening season and that this inflammation can be attenuated by the gut-specific anti-inflammatory drug mesalazine. Here, we manipulated the gut immune system of ground squirrels and diversity outbred (DO) mice with mesalazine, affected the microbiota with prebiotics, or combined both together. Mesalazine, prebiotics or both were provided through the diet to squirrels starting 48 hr after emergence from hibernation, and to DO mice after receipt of a gut microbiota transplant from ground squirrels at the same timepoint. Squirrels and mice stayed on their assigned treatment for up to 8 weeks, with tissues collected from a subset of animals after 4 and 8 weeks of treatment. We examined body mass, adiposity, glucose tolerance, and cytokines as markers of metabolic inflammation, hypothesizing that the combination of mesalazine and prebiotics would have the greatest effect. Body weight increased over time in all groups, whereas energy intake remained steady for mice and increased for squirrels. Adipocyte diameter increased in mesalazine/prebiotic treated squirrels over time. Glucose tolerance fluctuated over time and treatment in ground squirrels and mice, but prebiotic-treated animals tended to remain steady. In ground squirrel ileum, anti-inflammatory interleukin (IL)-10 increased over time and proinflammatory tumor necrosis factor (TNF)-α decreased in mesalazine-treated animals. Proinflammatory IL-6 increased over time in the prebiotic treatment ground squirrels. In DO mice, IL-10 was highest in animals receiving prebiotics, with or without mesalazine. In the colon of squirrels, prebiotics decreased TNF-α and increased IL-10 levels. A similar decrease in TNF-α was seen in prebiotic-treated mice. Omental WAT cytokines did not change in any of the animals or treatments. Together, our results indicate that prebiotics and mesalazine have separate effects on metabolic inflammation, and that their additive effect is not as strong as expected.

## 1 Introduction

Obesity and its associated comorbidities have become a worldwide epidemic. The driving force behind the widespread incidence of obesity is thought to be diets rich in fats, sugars, and highly-processed foods, as well as more sedentary lifestyles (Lingvay et al., 2024). Given the role of diet in the development of obesity, it is not surprising that the pathogenesis of the disease is thought to begin with the gut microbiota. Dietary changes lead to dysbiosis in the microbial communities which, in turn, increase stress and inflammation within the gut (Gil-Cardoso et al., 2017)(reviewed in (Scheithauer et al., 2020; Tilg et al., 2020). This subclinical intestinal inflammation can disrupt the gut barrier, leading to loss of barrier integrity and so-called “leaky gut”. The loss of gut barrier function allows the translocation of more bacterial products into the bloodstream and their transport to distant tissues (Amar et al., 2011). As a result, chronic inflammation develops throughout the body, particularly in highly metabolic tissues like adipose and liver, and this metabolic inflammation promotes the deposition of fat in these organs, leading to cellular dysfunction (Konrad and Wueest, 2014; Wu and Ballantyne, 2017; Xu et al., 2003).

Due to the inflammatory nature of obesity, anti-inflammatory drugs have been suggested as a possible treatment. Mesalazine (5-aminosalicylic acid or 5-ASA), a nonsteroidal anti-inflammatory drug (NSAID), has been used to treat inflammatory bowel diseases (IBD) for years (Rousseaux et al., 2005). It is not well-absorbed and consequently works locally on the gut (Mehta et al., 2023). These properties suggest that mesalazine is a potential treatment for metabolic inflammation. For example, mice on high-fat diets (HFD) experienced an anti-inflammatory shift within the colon and small intestine when treated with mesalazine for 16 weeks (Luck et al., 2015). In addition, our laboratory has shown that mesalazine reduced pro-inflammatory cytokines in the omental white adipose tissue (oWAT), ileum, and colon of fattening 13-lined ground squirrels (*Ictidomys tridecemlineatus*; (Zur Tulod et al., 2023)). Together, these studies suggest that mesalazine may be a viable treatment option to reduce the metabolic inflammation that is a hallmark for obesity.

Promotion of a healthy, stable bacterial community can also promote an anti-inflammatory state without the use of medication. Gut microbiota transfers (GMT) introduce microbial communities from another individual or species into the gut of a dysbiotic recipient and can, under the right conditions, change the community composition to favor a so-called healthy makeup. Cross-species GMT have demonstrated the potential role of the gut microbiota in fattening. For example, GMT from summer (lean) and hibernating (fattened) brown bears (*Urus arctos*) to mice showed that mice receiving summer microbiota stayed lean, whereas mice receiving hibernating microbiota gained mass (Sommer et al., 2016). In another study using germ-free recipients, GMT from obese mice induced more body and adipose mass than those receiving microbiota from lean mice (Ellekilde et al., 2014). These studies highlight the role of gut microbiota in the etiology of obesity and suggest GMT as a possible treatment.

GMT is an extreme example of therapies that can shift microbiota composition and function and is currently primarily used to treat recurring or recalcitrant *Clostridioides difficile* infections. A more common approach for altering microbial community composition is the use of dietary supplements such as prebiotics. Prebiotics are nondigestible plant compounds that offer a health benefit to the host via their effects on certain species within the microbiota (Bomhof et al., 2014). Some examples of prebiotics include pectin, inulin, lactulose, and oligofructose (Bomhof et al., 2014; Li et al., 2020). Pectins are isolated from the peels or pulp of fruits and vegetables, mostly sugar beets, apples, and citrus fruits and generally promote the growth of beneficial bacteria (Larsen et al., 2019). In fact, the prebiotic properties of pectins are thought to contribute to the anti-inflammatory properties of fruits and vegetables (Popov et al., 2013). Inulin is derived from vegetables, such as chicory root, asparagus, and onions. Inulin provides various health benefits, such as promoting mucin production, modulating microbiota composition, supporting a healthy immune system, and increasing mineral absorption and lipid metabolism (Qin et al., 2023; Swiech et al., 2025). Using prebiotics, such as pectin and inulin, with or without other pharmacological manipulations may be a beneficial avenue for treatment in obese individuals.

Most non-human studies addressing obesity use inbred mice that are either obesogenic knockout strains, such as *ob/ob* or *db/db* mice, or traditional inbred strains fed a high fat diet (HFD). While these mice are genetically consistent and offer less variability in results, they do not mirror the diversity of the human population well, and translational studies from these mice to humans have mixed success (Tuttle et al., 2018). Mammalian hibernators, such as ground squirrels, rapidly gain fat mass during their active season to prepare for hibernation, making them an excellent choice for studying rapid adiposity, like that seen in obesity. In addition to natural fat gain, wild-caught ground squirrels’ gut microbiotas have greater alpha and phylogenetic diversity in mucosal and luminal populations (Chiang et al., 2022). Thus, the microbiota diversity of ground squirrels is closer to humans than inbred, laboratory-raised lab mice.

For this study, we examined the effects of mesalazine, prebiotics, or a combination of mesalazine and prebiotics on body and adipose mass, caloric intake, glucose tolerance, and metabolic inflammation in thirteen-lined ground squirrels. Outbred mice were chosen as the non-hibernator model in this study because they have higher genetic, phenotypic, and environmental variability than inbred strains. By comparing outbred mice to thirteen-lined ground squirrels, we can compare responses to the treatments across species and determine which effects may be due to the hibernation phenotype. We hypothesize that the mesalazine/prebiotic treatment will have the greatest effect on these parameters in outbred mice and ground squirrels. This unique study can reveal new treatments for metabolic inflammation and obesity.

## 2 Materials and methods

### 2.1 Animals

Gravid female thirteen-lined ground squirrels were captured near the University of Wisconsin-Oshkosh (UWO) and brought into the UWO animal care facilities [as described in (Vaughan et al., 2006)]. After birth, dams and pups were housed in standard conditions (clear plastic bin with wire top, 43 cm W x 61 cm D x 20 cm H, 20-22 °C, 30-60% relative humidity, L:D cycle matching ambient conditions for Oshkosh, Wisconsin, USA) until pups were weaned. Squirrels were given PVC tubes for enrichment and hiding. To avoid handling stress, all squirrels throughout the study were transferred to new cages using the PVC tube (the handler would put a hand at each end during transfer, preventing the need for physical contact). Once weaned, pups were group-housed in the same conditions for 2 weeks before separating them into single housing in standard rat cages (25 cm W x 48 cm D x 20 cm H) until hibernation. Hibernation occurred in a hibernaculum (38-42°F, 24 hours dark) for at least 4 months. On April 2, 2024, 25 male and 39 female yearling ground squirrels were moved to the warm room (forced emergence), singly housed, and kept at 12:12 L:D cycle. Treatments began 48 hours after emergence. Animals were fed Teklad Global 18% Protein Rodent Diet (Inotiv, Madison, WI, USA) base diet *ad libitum* along with 5 g (1 tbsp) of sunflower seeds weekly. Leftover food and sunflower seeds were weighed during weekly cage cleaning and subtracted from total grams given. Total kilojoules of energy consumed were calculated using nutrient information provided by the diet manufacturer. The mass of squirrels was calculated weekly during cage cleaning by taking a clean cage on a scale, then adding the squirrel to the cage.

Diversity outbred (DO) mice (n=64, 32 males & 32 females, 4 weeks old) were purchased from Jackson Laboratories (Bar Harbor, ME, USA). Mice were separated by sex and added to standard mice cages (n=2 mice/cage; 18 cm W x 29 cm D x 13 cm H). Temperature and relative humidity and L:D cycle (12:12) were identical to that of ground squirrels. Mice were given paper Bio-Huts (Bio-Serv, Flemington, NJ USA) for nesting boxes. Animals were fed Teklad Global 18% Protein Rodent Diet (Envigo, Madison, WI, USA) base diet *ad* libitum along with 3 g (1 tsp) of sunflower seeds given weekly. Energy intake was calculated as described for ground squirrels above.

All protocols and procedures for this study were approved and overseen by the University of Wisconsin-Oshkosh Institutional Animal Care and Use Committee (IACUC; protocol #330).

### 2.2 Microbiota transfer

In 2022, yearling ground squirrels (n = 6, 3 male and 3 female) were aroused from hibernation, moved to a warm room, and fed Teklad diet as described above. Forty-eight hr after emergence from hibernation, squirrels were anesthetized with 5% isoflurane and euthanized via guillotine. The gut was tied off with a suture from the last 8 cm of the ileum through the first 4 cm of the colon, placed in sterile 1X PBS, and moved to an anaerobic chamber filled with 90% N_2_ and 10% CO_2_. In the chamber, the gut was cut open and contents were removed with sterile instruments. Gut mucosa was also removed by scraping the mucosal surface with sterile glass slides. All gathered material was combined, mixed, and split into 2 sterile serum vials containing brain-heart infusion broth supplemented with 0.5 mg/ml L-cysteine and 20% glycerol. The total amount of time for each extraction was no more than 30 minutes. Contents were slowly frozen to maintain viability of bacteria (4 °C for 2 hours, -20 °C for 12 hours, and then -80˚C for storage).

Mice were acclimated to the UWO Animal Facilities for 1-week prior to beginning antibiotic treatment (vancomycin [0.5g/L], neomycin [1g/L], ampicillin [1g/L], metronidazole [1g/L]) for 3 days in drinking water (Regan et al., 2026; Yoshiya et al., 2011). Mice were then returned to fresh water for 48 hours. Prior to gut microbiota transfer, food was removed and the mice received polyethylene glycol (PEG) solution (0.2 mL/dose, by mouth) three times, 20-minutes apart (Wrzosek et al., 2018). Four hours after the last PEG treatment, mice were gavaged with the ground squirrel microbiota mixture (100 μl; collection described in the previous paragraph). Mice were given a second microbiota gavage of the same material 1 week after the initial gavage. Prior to gavage, an aliquot of the mixture was stored at -80 °C for sequencing analysis.

### 2.3 Treatments

Yearling ground squirrels (n=63) were separated into 4 treatment groups, control (n=15, 6 males and 9 females), mesalazine (n=16, 6 males and 10 females), prebiotic (n=16, 6 males and 10 females), and mesalazine/prebiotic (n=16, 7 males and 9 females). Treatments began 48 hours post-emergence and were formulated in the diet on the Teklad 2018 background. These included mesalazine diet (1% wt/wt, equivalent to ~500 mg/kg dose per day), prebiotics diet (3% wt/wt inulin and 3% wt/wt pectin), and mesalazine/prebiotic mixture (with previous formulations combined). The control group remained on the Teklad base diet. Treatments lasted for 8 weeks.

Following microbiota transfer, DO mice were separated into the same 4 treatment groups as described for ground squirrels, with equal number males to females (n = 16, 8 males and 8 females per treatment group). Diet formulations for prebiotics were identical, but the formulation for mesalazine was different (0.31%, still equivalent to ~500 mg/kg dose per day) because of differences in body mass and food intake between ground squirrels and mice. Treatments lasted for 8 weeks.

Mesalazine was purchased from Cayman Chemical (Ann Arbor, MI, USA). Inulin (> 90% pure) and pectin (with ~74% galacturonic acid, sourced from citrus) were provided by Inotiv (Madison, WI, USA).

### 2.4 Glucose tolerance tests

GTT for ground squirrels and mice were conducted during weeks 1, 3, 5, and 7 of treatment. Squirrels (n = 2 males & n = 2 females per treatment group) and DO mice (n = 3 males & n = 3 females per treatment group) were selected from available animals each time and fasted 5 hours prior to GTT. Squirrels were anesthetized for the GTT procedure (induction with 5% isoflurane, maintenance with 4% isoflurane) because of their wild, non-docile nature, but mice were not because of their sensitivity to anesthetics. For ground squirrels, the tail artery was pierced with a 27G needle (1/2”) for blood collection. Mice were placed on the wire cage lid with their tail hanging over the ledge. To minimize stress and biting during handling, a cardboard divider with a notch cut out for the tail was utilized to separate the mouse body from the handler. A sterile razor blade was used to nick the side of the tail to collect a drop of blood from the tail vein.

A baseline glucose reading was taken for both species using a Contour Next Glucometer. Following the baseline reading, both squirrels and mice were injected i.p. with 1g/kg dextrose, and blood collected at 15 minutes and 60 minutes post-injection. Squirrels remained under sedation until the 15-minute post-injection blood collection, after which the squirrels were taken off isoflurane and allowed to recover and drink water. Squirrels were briefly anesthetized again for blood collection at 60-minutes post-injection. After the procedure was finished, all animals were returned to normal housing and food was returned.

Due to veterinary requirements at the University of Wisconsin Oshkosh, animals had to have a 2-week resting period between GTTs; therefore, the same animal could not be used for 2 successive tests. We also wanted to include males and females in GTT data at each timepoint. Together, these two criteria limited the number of animals that were used for each GTT, making sample sizes smaller than preferred.

### 2.5 Tissue collection

After 4 and 8 weeks of treatment, half the ground squirrels (n = 3–4 male, 4–5 female) and mice (n = 2–4 male, 3–4 female) from each treatment group were euthanized. Animals were anesthetized with 5% isoflurane then weighed on a tared scale to record body weight. Squirrels were euthanized by rapid decapitation with a guillotine while sedated, whereas mice were euthanized by cervical dislocation while sedated. Ileum, cecum, and colon were removed in their entirety. Cecum and colon contents were removed with sterile forceps and spatulas. Forceps and spatulas were sterilized with a bead sterilizer between animals. The remaining ileum and colon tissue was flash frozen in liquid nitrogen. Liver, intra-abdominal WAT (iaWAT), and omental WAT (oWAT) were removed in their entirety and weighed. A portion of oWAT was fixed in 10% neutral buffered formalin and the remainder was flash frozen in liquid nitrogen. Tissues were stored at -80 °C until analysis.

### 2.6 Adipocyte diameter measurements

oWAT tissues from ground squirrels and mice were fixed in 10% neutral buffered formalin for 5 days and then stored in 70% ethanol until processing by the Histology Core at the University of Wisconsin Madison School of Veterinary Medicine (Madison, WI, USA). Samples were processed by embedding in paraffin and sectioning (7 um), followed by staining with hematoxylin and eosin. Images of each sample were captured with a 10x objective lens (100x magnification) using CellSens software (Evident Scientific, Waltham, MA). In order to randomly select adipocytes for measurements, the following methods were utilized: At 10x objective magnification, three 2.50 x 1.25-mm images were analyzed. Adipocytes were selected at x (horizontal), and y (vertical) coordinates ([1.0, 1.0], [1.3, 0.7], [1.7, 0.3], [2.0, 0.1]). A diameter measurement was taken on the Y and X axis of each cell. These values were averaged to determine an average diameter measurement of each randomly selected adipocyte. The measuring procedure was slightly modified for the mouse oWAT diameter measurements due to a greater scarcity of cells. The coordinates of the measured cells were changed to (1.00, 0.76), (1.00, 0.80), (1.00, 0.84), (1.00, 0.88). Of the 58 mouse oWAT slides, 13 did not have enough adipocytes for the randomization method to work correctly. In these situations, every other adipocyte was selected. Seven of the 58 mouse oWAT slides (2 controls, 2 mesalazine-treated, and 3 prebiotic-treated mice) were unmeasurable because no measurable adipocytes were present in the section.

### 2.7 ELISA

Enzyme-linked immunosorbent assays were completed for interleukin (IL)-6, IL-10, and tumor necrosis factor (TNF)-α. Tissue was weighed (50–75 mg of ileum and colon, 100–150 mg of oWAT) and homogenized in 1X PBS containing Halt protease inhibitor cocktail (Thermo Scientific, Waltham, MA, USA). oWAT was manually homogenized with a Dounce homogenizer, while ileum and colon samples were mechanically homogenized with a Tissue Master 125 (OMNI International, Lake Villa, IL). Homogenized samples were centrifuged (10,000 x g, 10 minutes, 4 °C) and the supernatants were used as samples in the ELISA. For ground squirrel samples, rat-specific ELISA kits were used because rat shows the closest homology to thirteen-lined ground squirrels (> 95% protein similarity for these cytokines; (Sonsalla et al., 2021)). Mouse-specific ELISA kits were used for mouse samples. All ELISA kits were purchased from BD Biosciences (San Jose, CA) and potential cross-reactivity is listed on the manufacturer website. ELISA results were normalized to protein concentrations determined using BCA protein assays (Pierce, Life Technologies, Waltham, MA) of the supernatants used in the ELISA.

### 2.8 Microbiota analysis

DNA extraction and sequencing were conducted as described in (Grond et al., 2021). Briefly, DNA was extracted from mouse cecum and colon lumen contents and the squirrel microbiota mixture (used for gavage) using the Qiagen RNeasy PowerMicrobiome Kit (Qiagen, Hilden, Germany) according to manufacturer’s protocols except the DNase digestion step was omitted. DNA concentration and purity were measured with a Qubit Fluorometer (Invotrogen, Carlsbad, CA), and the V4 region of the 16S rRNA gene PCR-amplified (in triplicate) using universal bacterial primers 515 F (5’-GTGYCAGCMGCCGCGGTAA-3’) and 806 R (5’-GGACTACNVGGGTWTCTAAT-3’) with attached 12-bp Golay barcodes (Earth Microbiome Project protocol; https://earthmicrobiome.ucsd.edu/ (Thompson et al., 2017). PCR products were pooled and cleaned using the AxyPrep Mag PCR cleanup kit (Axygen, Union City, CA). Final concentrations were determined (Kapa Library Quantification Kit; Roches Sequencing Solutions Inc., Pleasanton, CA), samples combined (equimolar), and libraries sequenced (paired-end; 300 x 2bp) using the Ilumina MiSeq platform (MiSeq v2 Reagent Kit). Sequences are available at NCBI under BioProject # PRJNA1494925.

Sequences were processed using QIIME 2 (Bolyen et al., 2019). Sequences were trimmed and filtered, chimeras removed and taxonomically classified to the genus level using the Silva database (v.138.1). Those identified as chloroplasts and mitochondrial, and those that did not align to bacteria, were removed, and a phylogenetic tree generated of the microbial amplicon sequence variants (ASVs). Samples were rarified to 5,080 sequences/sample; all samples with lower read numbers had fewer than 634 reads/sample. We plotted the relative abundances of the top 10 phyla and top 20 genera (ggplot2 package; v.4.0.1; (Wickham, 2016)).

### 2.9 Statistical analysis

Statistical analyses for all but microbial analyses were performed using GraphPad Prism software (San Diego, CA). Body mass and energy intake were analyzed using repeated measures ANOVA. GTT, weighed tissues, and ELISA were analyzed for outliers prior to normality tests (Shapiro-Wilk, Kolmogorov-Smirnov, and Anderson-Darling). If most normality tests did not pass, the data was transformed logarithmically. GTT was analyzed by calculating the area under the curve (AUC) for each test. AUC and ELISA were analyzed by 2-way (time, treatment) or 3-way (time, treatment, sex) ANOVAs with *post-hoc* comparisons (Tukey’s Honest Significant Difference). Differential abundances (log 2-fold change) of taxa in mice microbiotas were compared among treatments within post-gavage time points and within treatments between post-gavage time points using the ALDEx2 package in R. Due to volume limitations, we sequenced the squirrel microbiota mixture used for gavage rather than the individual samples collected from each squirrel prior to mixing; therefore, we were unable to statistically compare the composition of the squirrel microbiota mixture to that of the recipient mice. Any results with p-values ≤ 0.05 were considered significant.

## 3 Results

### 3.1 Body mass

**Figure 1:**
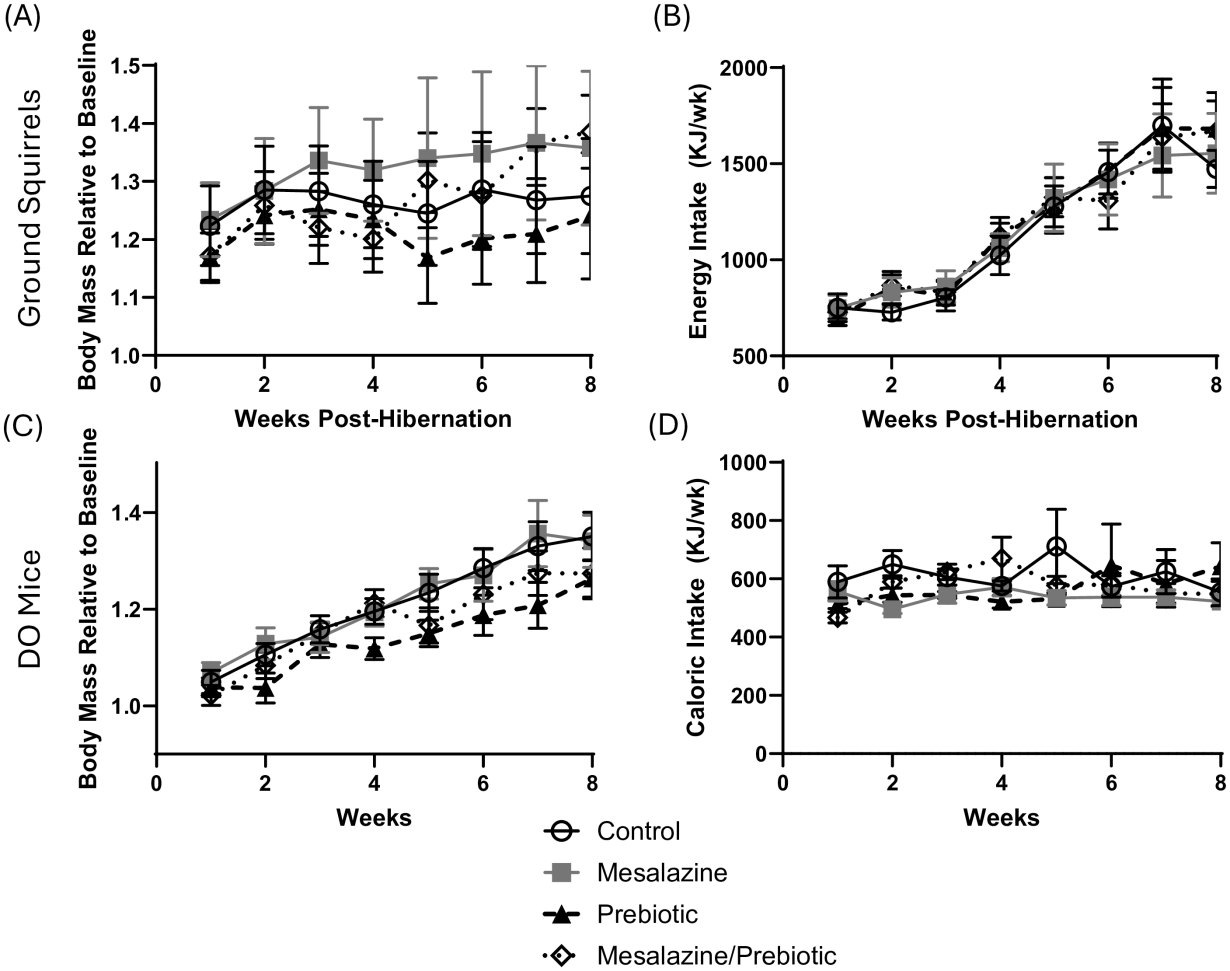
Body mass and energy intake in ground squirrels and mice. **(A)** Ground squirrel body mass relative to pre-treatment mass (n= 8-16/group/timepoint). **(B)** Ground squirrel energy intake (n=8-16/group/timepoint). **(C)** DO mouse body mass relative to pre-treatment mass (n= 5-16/group/timepoint). **(D)** DO mouse energy intake (n=5-16/group/timepoint).

Ground squirrels and mice were weighed weekly for 9 weeks, starting one week before treatment began. Squirrel body mass significantly increased from baseline during the 8-week treatment period but was not significant between treatments (**Figure 1A**; time p < 0.0001, treatment p > 0.05, time x treatment p > 0.05). Body mass was greater in male squirrels compared to female squirrels in all treatment groups (sex p < 0.05), but mass of male squirrels tended to level off earlier than that of females, who continued to gain weight over the entire treatment period (time * sex p < 0.0001, data not shown). Mouse mass significantly increased relative to baseline over 8 weeks but was also not significant between treatments (**Figure 1C**; time p < 0.0001, treatment p > 0.05, time x treatment p > 0.05). There was no difference in mouse mass by sex (sex p > 0.05, data not shown).

**Figure 2:**
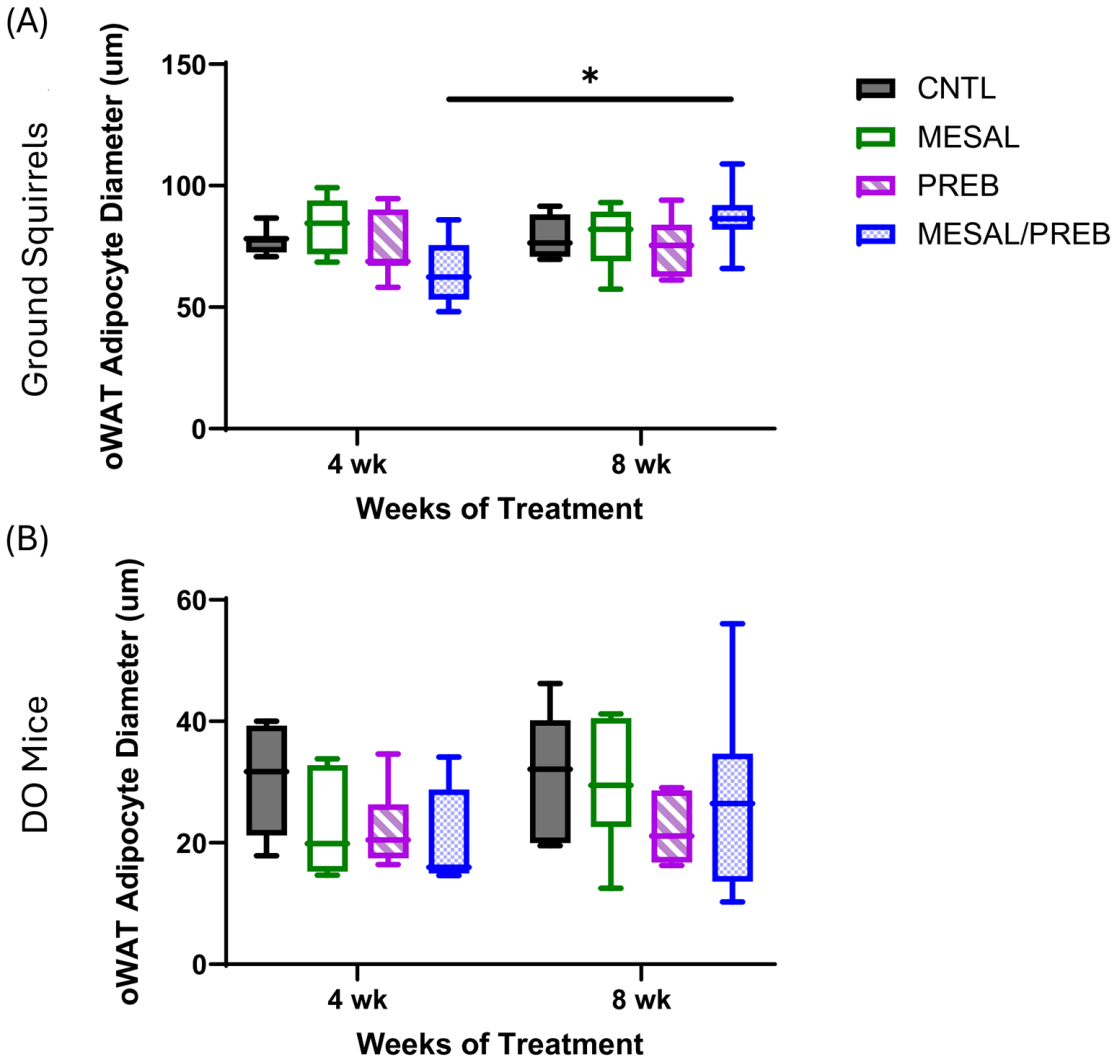
Adipocyte diameter in ground squirrels and mice. **(A)** Ground squirrel oWAT adipocyte diameter measured histologically (n= 6-8/group/timepoint). **(B)** DO mouse oWAT adipocyte diameter measured histologically (n= 5-8/group/timepoint). *P ≤ 0.05.

We examined masses of the liver and two WAT depots (iaWAT and oWAT). When both sexes were combined, treatment and time had no effect on the mass of liver, iaWAT, or oWAT in either species (**Table 1**; all p-values > 0.05). When comparing liver mass in squirrels between sexes, males tended to have greater liver mass compared to females in the control, mesalazine, and prebiotic treatment groups (sex p < 0.05, **Supplementary Table 1**). There was no difference in liver mass between sexes for mesalazine/prebiotic treatment in squirrels. Neither sex nor treatment had any effect on liver mass in mice (all p-values > 0.05). Neither sex nor treatment had any effect on iaWAT mass in squirrels (all p-values > 0.05). In mice, iaWAT mass tended to be greater in males than females in the mesalazine/prebiotic treatment group (sex p < 0.05, **Supplementary Table 2**). Neither sex nor treatment had any effect on oWAT mass in squirrels or mice (all p-values > 0.05). We examined oWAT adipocyte diameter as a measure of hypertrophy. In squirrels, adipocyte diameter increased in the mesalazine/prebiotic group over the study period (**Figure 2A**, time x treatment p = 0.0153). This increase was mainly due to an increase in adipocyte diameter in female squirrels (**Supplementary Figure 1A**, time x sex p = 0.0015, time x treatment x sex p = 0.0054). In mice, no differences in adipocyte diameter were seen when the sexes were combined (**Figure 2B**, p > 0.05); however, when compared by sex, females treated with mesalazine/prebiotic diet tended to have smaller adipocytes than control females and males in the same treatment group (**Supplementary Figure 1B**, treatment x sex p = 0.0453).

**Table 1:** Tissue mass.

	LIVER (g)				iaWAT (g)				oWAT (g)			
	TLGS		DO Mice		TLGS		DO Mice		TLGS		DO Mice	
	4WK	8WK	4WK	8WK	4WK	8WK	4WK	8WK	4WK	8WK	4WK	8WK
Control	6.40 ± 1.26 (n=7)	6.15 ± 1.20 (n=8)	1.03 ± 0.23 (n=6)	1.17 ± 0.56 (n=6)	4.34 ± 1.48 (n=7)	5.71 ± 2.34 (n=8)	0.36 ± 0.22 (n=6)	0.68 ± 0.58 (n=6)	1.34 ± 0.58 (n=7)	1.63 ± 0.47 (n=8)	0.16 ± 0.01 (n=6)	0.38 ± 0.52 (n=6)
Mesalazine	7.15 ± 2.67 (n=8)	6.22 ± 2.17 (n=8)	1.03 ± 0.43 (n=8)	1.21 ± 0.15 (n=7)	6.17 ± 3.08 (n=8)	5.54 ± 3.48 (n=8)	0.35 ± 0.20 (n=8)	0.52 ± 0.43 (n=7)	1.60 ± 0.64 (n=8)	1.69 ± 0.95 (n=8)	0.34 ± 0 = .53 (n=8)	0.15 ± 0.04 (n=7)
Prebiotic	5.64 ± 1.07 (n=8)	6.16 ± 1.49 (n=8)	1.01 ± 0.40 (n=7)	1.10 ± 0.43 (n=8)	3.09 ± 0.87 (n=8)	4.45 ± 1.95 (n=8)	0.36 ± 0.19 (n=7)	0.44 ± 0.26 (n=8)	1.09 ± 0.20 (n=8)	1.39 ± 0.52 (n=8)	0.27 ± 0.28 (n=7)	0.25 ± 0.27 (n=8)
Mesalazine/Prebiotic	6.04 ± 1.38 (n=8)	7.55 ± 2.70 (n=7)	1.33 ± 0.28 (n=8)	1.40 ± 0.25 (n=8)	4.14 ± 1.81 (n=8)	6.42 ± 4.35 (n=7)	0.28 ± 0.18 (n=8)	0.77 ± 0.77 (n=8)	1.65 ± 0.71 (n=8)	1.80 ± 0.58 (n=7)	0.16 ± 0.04 (n=8)	0.17 ± 0.04 (n=8)

Means for liver, iaWAT, oWAT, of TLGS and mice separated by 4 and 8 weeks, and treatment with standard error. TLGS, thirteen-lined ground squirrel.

### 3.2 Energy intake

Energy intake for squirrels increased slowly in the first 3 weeks of treatment and then increased more dramatically until 8 weeks (**Figure 1B**; time p < 0.0001). There was no difference between treatments (treatment p > 0.05, time x treatment p > 0.05), and there were no sex differences in squirrels (sex p > 0.05). Mouse energy intake did not vary over time or by treatment, but the interaction of the two was significant due to some week-by-week variability among groups (**Figure 1D**; time p > 0.05, treatment p > 0.05, time x treatment p = 0.0088). Male mice in the control, mesalazine, and prebiotic groups, but not the mesalazine/prebiotic group, ate more than females (sex p < 0.01, data not shown).

### 3.3 Glucose tolerance test

**Figure 3:**
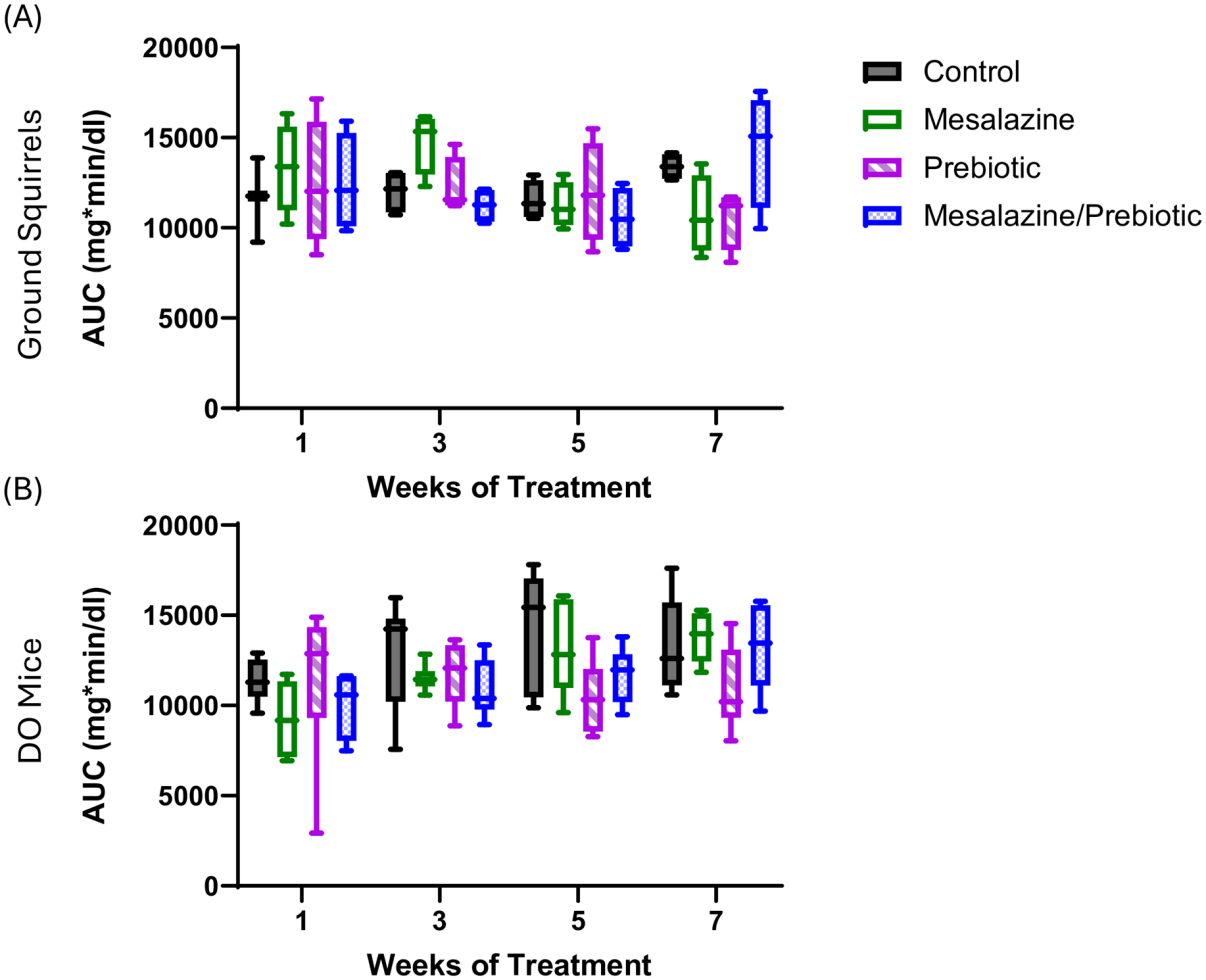
Glucose tolerance during treatment. **(A)** Ground squirrel glucose tolerance as measured by the area under the curve (AUC) (n= 4/group/timepoint). **(B)** DO mouse glucose tolerance (n=4-6/group/timepoint).

The glucose tolerance of squirrels was not affected by time or treatment alone, but there was variation between treatment groups over time with decreases in the mesalazine group at 5 and 7 weeks, in the prebiotic group at 7 weeks, and in the mesalazine/prebiotic group at 3 and 5 weeks (**Figure 3A**; time p > 0.05, treatment p > 0.05, time x treatment p = 0.0174). Squirrel glucose tolerance did not differ by sex (sex p > 0.05, data not shown). Glucose tolerance in mice generally increased over the treatment period but was not affected by treatment (**Figure 3B**; time p= 0.0194, treatment p > 0.05, time x treatment p > 0.05). In mice, males in the control, mesalazine, and mesalazine/prebiotic groups, but not the prebiotic group, had lower tolerance (higher AUC) than females (**Supplementary Figure 2**, sex p < 0.01).

### 3.4 Ileum cytokines

**Figure 4:**
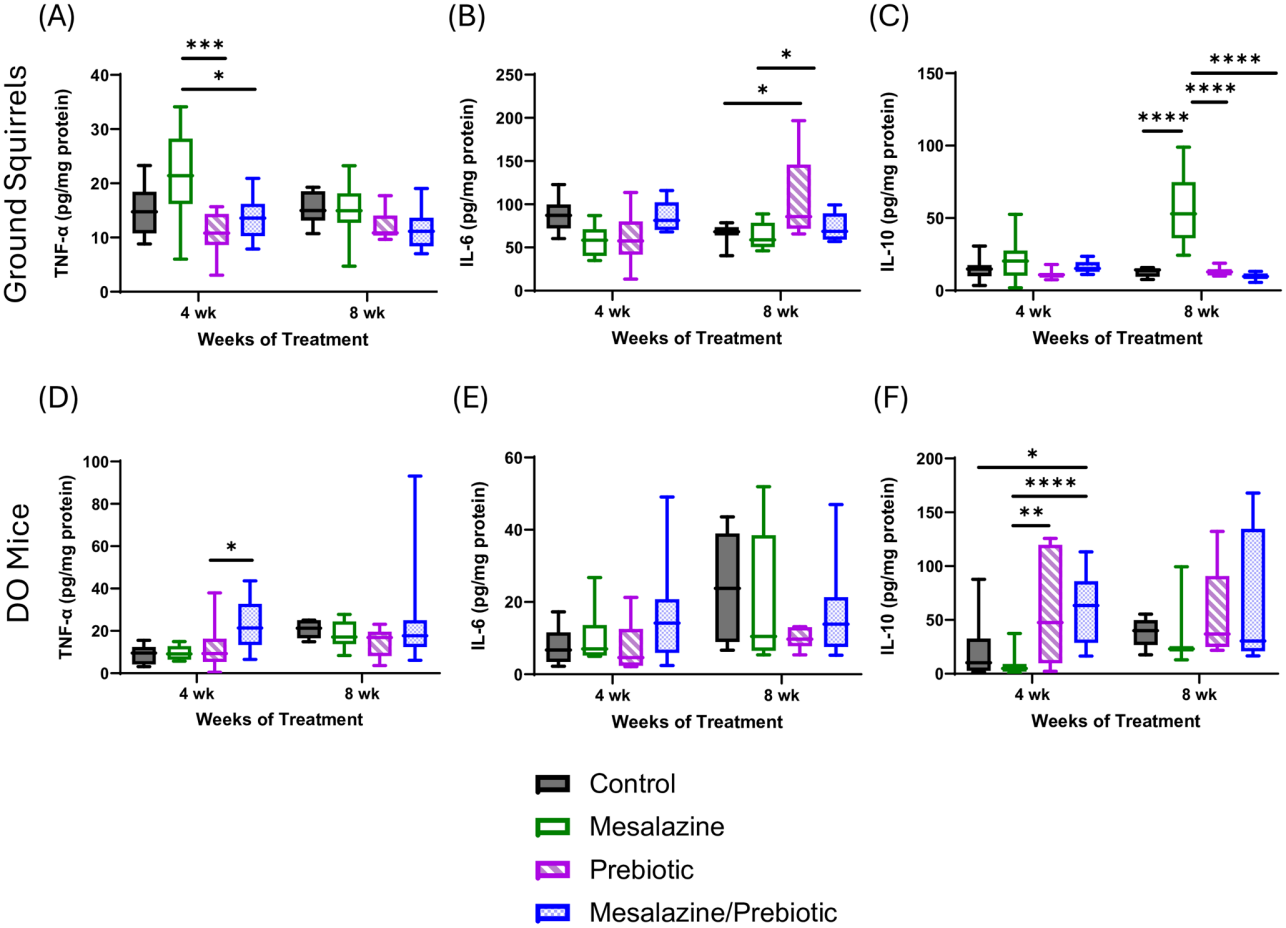
Cytokine levels in the ileum of ground squirrels and mice during treatment. Ground squirrel levels of **(A)** TNF-α, **(B)** IL-6, and **(C)** IL-10 in ileum tissue (n= 8-16/group/timepoint). DO mouse levels of **(D)** TNF- α, **(E)** IL-6, and **(F)** IL-10 in ileum tissue (n = 6-8/group/timepoint). *P ≤ 0.05, **P < 0.01, ***P < 0.001, ****P < 0.00001.

TNF- α levels were lower in squirrels receiving prebiotic treatment with or without mesalazine compared to mesalazine alone after 4 weeks (**Figure 4A**; time p > 0.05, treatment p < 0.05, time x treatment p > 0.05). After 8 weeks of treatment, levels in the mesalazine treatment group dropped to match the other groups. There was a difference between TNF- α expression between sexes in the mesalazine/prebiotic treatment, with female levels higher than males (**Supplementary Figure 3A**, sex p < 0.005). TNF- α levels were generally low in mice, although mice given mesalazine/prebiotic treatment had higher levels than prebiotic treated mice at 4 weeks (**Figure 4D**; time p = 0.0081, treatment p = 0.0588, time x treatment p > 0.05). There were no differences between the sexes in mice (sex p > 0.05, data not shown). IL-6 levels in prebiotic-treated squirrels were higher than control and mesalazine after 8 weeks of treatment (**Figure 4B**; time p > 0.05, treatment p = 0.0726, time x treatment p = 0.0044). In mice, IL-6 generally increased between the 4- and 8-week tissue collections, although the increase is not apparent in prebiotic-treated mice (**Figure 4E**; time p = 0.0065, treatment p > 0.05, time x treatment p > 0.05). There were no differences in IL-6 concentrations between sexes in either species (sex p > 0.05, data not shown). IL-10 levels increased in mesalazine-treated squirrels between 4 and 8 weeks of treatment and levels at 8 weeks were higher than all other groups (**Figure 4C**; time p = 0.0258, treatment p < 0.0001, time x treatment p < 0.0001). There were no differences in IL-10 between sexes in squirrels (sex p > 0.05, data not shown). In mice, IL-10 levels after 4 weeks of treatment were highest in the groups receiving prebiotics, with or without mesalazine (**Figure 4F**; time p = 0.0024, treatment p = 0.0004, time x treatment p = 0.0324). There was a difference between sexes in the prebiotic treatment, with female mice tending to produce more IL-10 than males (**Supplementary Figure 4A**, sex p < 0.05).

### 3.5 Colon cytokines

**Figure 5:**
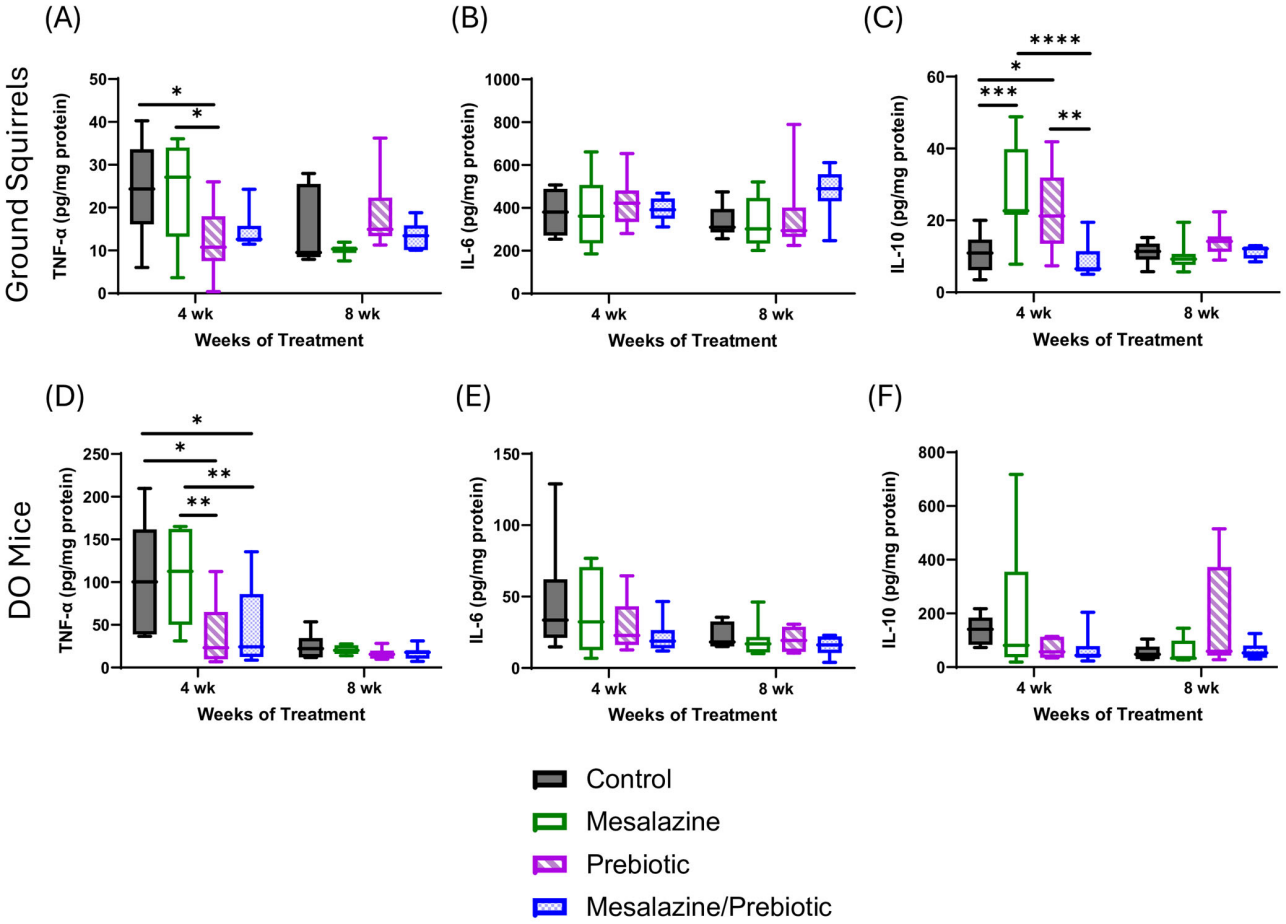
Cytokine levels in colon of ground squirrels and mice during treatment. Ground squirrel levels of **(A)** TNF-α, **(B)** IL-6, and **(C)** IL-10 in colon tissue (n= 8-16/group/timepoint). DO mouse levels of **(D)** TNF- α, **(E)** IL-6, and **(F)** IL-10 in colon tissue (n = 6-8/group/timepoint). * <= 0.05; ** < 0.01; *** < 0.001; **** < 0.0001.

After 4 weeks of treatment, colon TNF- α levels in squirrels were lowest in the prebiotic treatment group (**Figure 5A**; time p = 0.0335, treatment p > 0.05, time x treatment p = 0.0052). This was mainly due to low levels in prebiotic-treated females (**Supplementary Figure 3B**). There were no differences between treatment groups after 8 weeks. In mice, TNF-α levels were also lowest in prebiotic-treated animals, with or without mesalazine, and the levels decreased in all groups after 8 weeks of treatment (**Figure 5D**; time p < 0.0001, treatment p = 0.0019, time x treatment p = 0.0829). The changes after 4 weeks in mice varied by sex, with prebiotic treatment decreasing TNF-α levels in female mice, while mesalazine/prebiotic treatment had a larger effect on males (**Supplementary Figures 4C, D**). IL-6 levels did not change in squirrel colon by time or treatment (**Figure 5B**). IL-6 levels in mice were lower at 8 weeks compared to 4 weeks for all treatments (**Figure 5E**; time p = 0.0064, treatment p > 0.05, time x treatment p > 0.05). There were no differences in IL-6 by sex (data not shown). Mesalazine and prebiotic treatment alone, but not together, increased IL-10 levels in squirrels after 4 weeks of treatment (**Figure 5C**; time p = 0.0872, treatment p = 0.0014, time x treatment p = 0.0008). This effect did not last through 8 weeks of treatment. IL-10 levels in mice were not affected by any treatment or time (**Figure 5F**; time p > 0.05, treatment p > 0.05, time x treatment p > 0.05). There was high variability in IL-10 levels in mesalazine-treated mice after 4 weeks, likely due to a dramatic and significant difference between the sexes with a large increase in IL-10 in female mice at this timepoint (**Supplementary Figure 4B**, time p = 0.0157, time x treatment x sex p = 0.0321).

### 3.6 oWAT cytokines

**Figure 6:**
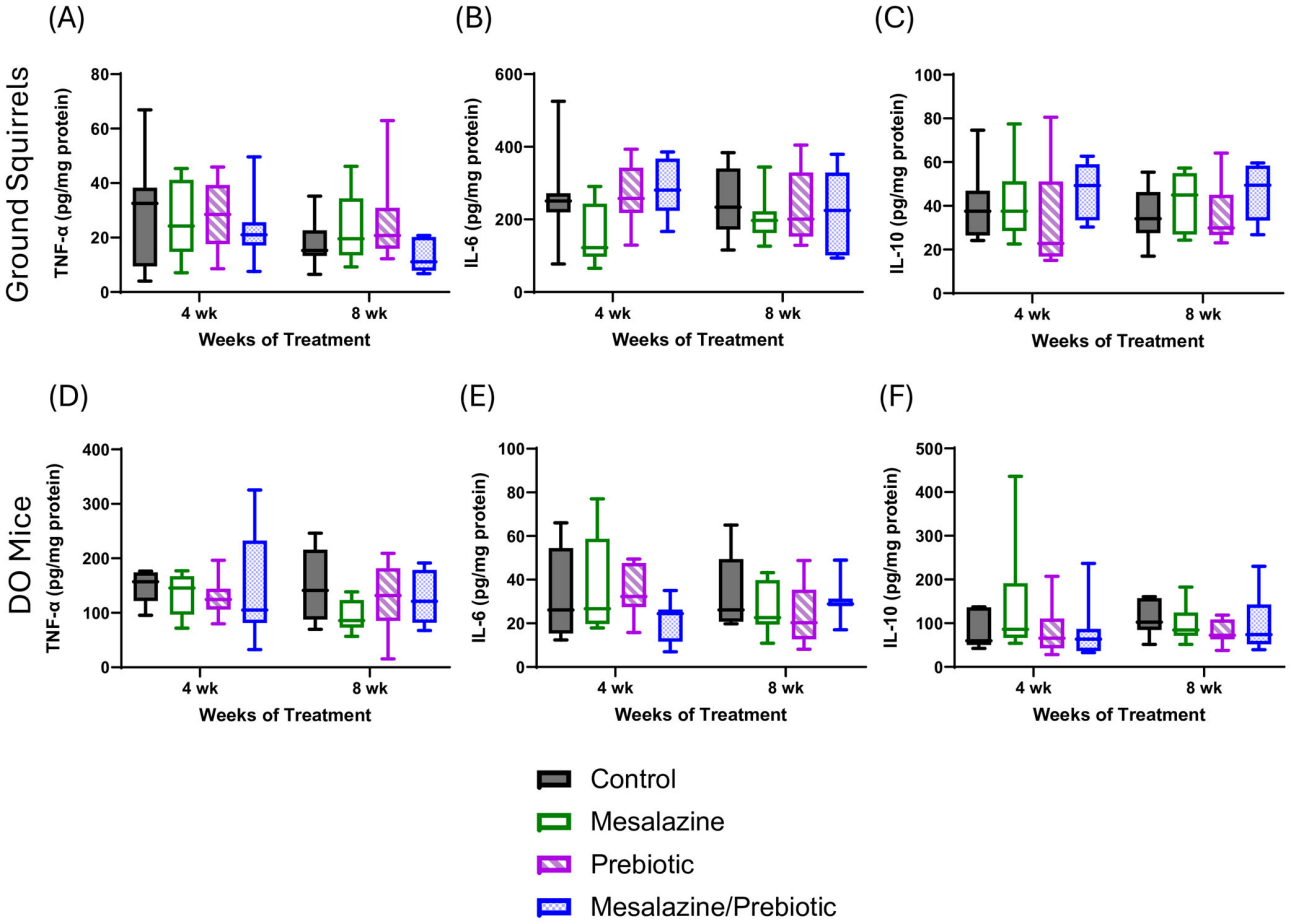
Cytokine levels in oWAT of ground squirrels and mice. Ground squirrel levels of **(A)** TNF-α, **(B)** IL-6, and **(C)** IL-10 in omental white adipose tissue (n= 8-16/group/timepoint). DO mouse levels of **(D)** TNF- α, **(E)** IL-6, and **(F)** IL-10 in omental white adipose tissue (n = 6-8/group/timepoint).

When both sexes were combined, neither treatment nor time had any effect on levels of TNF-α (**Figures 6A, D**), IL-6 (**Figures 6B,E**), or IL-10 (**Figures 6C, F**) in either species (all p-values > 0.05). When examined by sex, male squirrels treated with prebiotics, with (time x sex p = 0.0076) or without mesalazine (time x sex p = 0.0096), decreased IL-6 levels between the 4 wk and 8 wk timepoints (**Supplementary Figures 3C, D**). In contrast, female mice treated with mesalazine (sex p = 0.0409) or prebiotic (treatment x sex p = 0.0419) separately but not together decreased IL-6 levels with treatment compared to controls (**Supplementary Figures 4E, F**).

### 3.7 Microbial community composition

**Figure 7:**
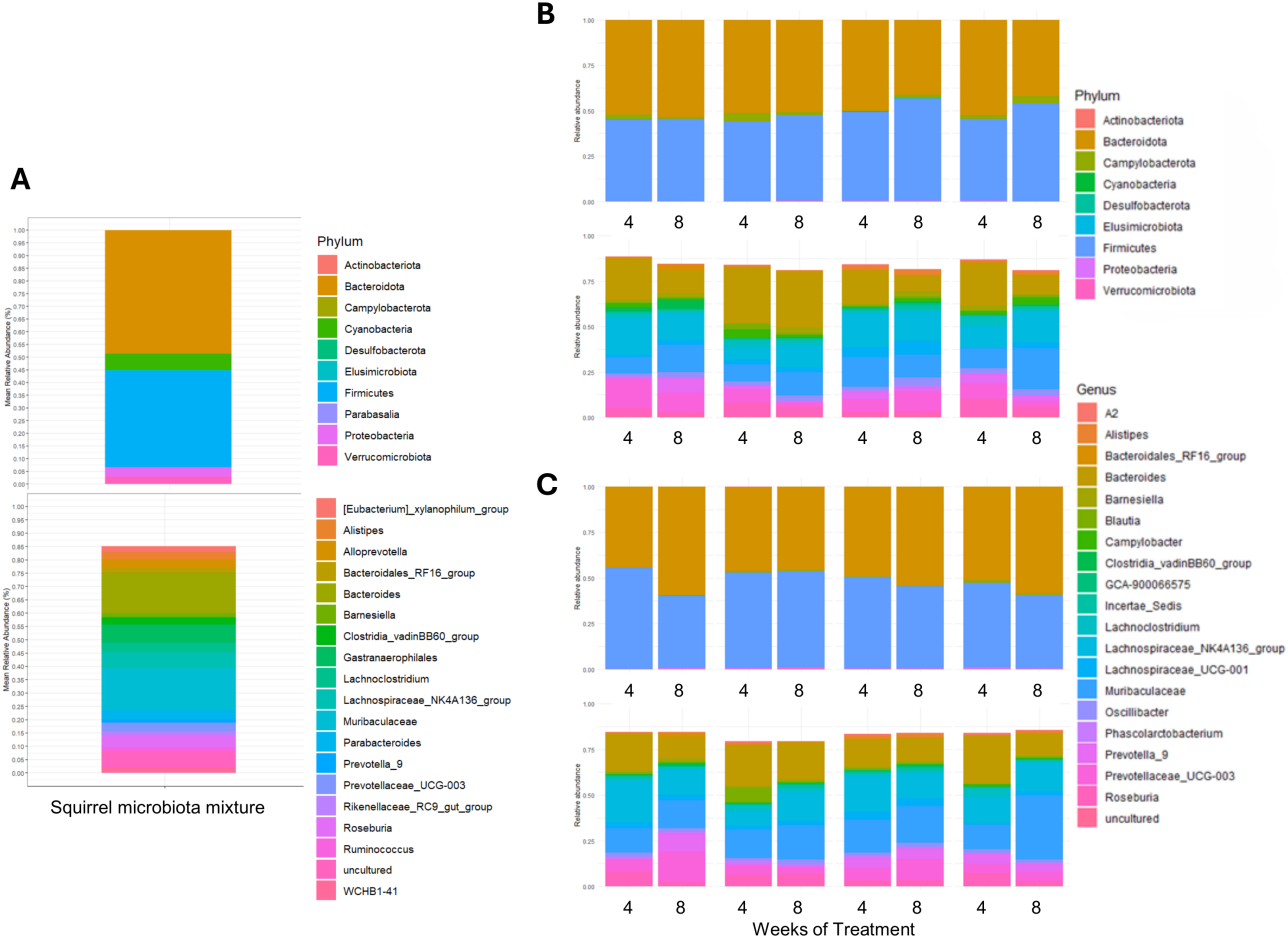
Relative abundances of top 10 phyla and top 20 genera. **(A)** Ground squirrel microbiota mixture gavaged into mice arranged by phylum (top) and genus (bottom). **(B)**. Cecum lumen contents of control and treatment mice collected 4 weeks and 8 weeks post-gavage arranged by phylum (top) and genus (bottom). **(C)** Colon lumen contents of control and treatment mice collected 4 weeks and 8 weeks post-gavage arranged by phylum (top) and genus (bottom).

We sequenced 116 samples from the cecum and colon of mice, losing 1 sequence upon rarefaction. Bacteroidota (48.6%) and Firmicutes (37.8%) were the dominant phyla in the gavage mixture (**Figure 7A**), followed by Proteobacteria (3.8%) and Verrucomicrobiota (2.9%). None of the remaining top 10 phlya were present above 1% percent in the mixture. Eight total phyla were found in the cecal lumen microbiotas of mice (**Figure 7B**). The dominant phyla in the cecum lumen microbiotas of recipient mice on the control diet were Bacteroidota (52.9%) and Firmicutes (45.2%). Of the remaining phyla, only Campylobacterota was above 1%. Similar abundances were found in the cecum lumen microbiotas of mice on all three diet treatments (**Figure 7B**). Nine phyla were observed in the colon lumen microbiotas of mice (**Figure 7C**). The dominant phyla in the control mice were Bacteroidota (51.7%) and Firmicutes (47.4%). Similar results were observed in treatment mice, and like in the cecum lumen, the only other phylum present above 1% was Campylobacterota (**Figure 7C**). The genus *Bacteroides*, and an unidentified genus in the family Muribaculaceae, which were the most abundant in the gavage sample, were consistently among the top four abundant genera in the cecum and colon lumen microbiotas of control and treatment mice (**Figures 7B, C**).

No phyla were differentially abundant within or among treatments in either cecum lumen or colon lumen microbiotas. At the genus level, within treatment comparisons showed that at 4-weeks post gavage, the genera *Campylobacter* and *Erysipelatoclostridum* were significantly enriched in the cecal microbiotas of mice treated with mesalazine/prebiotic compared to those treated only with mesalazine (**Supplementary Table 3**). Additionally, *Campylobacter* and *Anaerostipes* were significantly enriched in the colon microbiotas of mice treated with mesalazine/probiotic vs mesalazine only (**Supplementary Table 3**). Within colon microbiotas of recipient mice 4-weeks post gavage, Clostria_*UCG-014* was enriched in control mice compared to those fed a mesalazine diet, and the genera *Bacteroides* and *Campylobacter* were enriched in mice treated with mesalazine vs prebiotic (**Supplementary Table 3**). At 8-weeks post gavage, the microbiotas of mice treated with prebiotic alone were enriched in *Anaerotruncus c*ompared to those treated with mesalazine/prebiotic mixture. When comparing within treatments, Lachnospiraceae_UGC-001 and an uncultured Firmicutes were enriched at 4-weeks post gavage in the cecal microbiotas of mice treated with prebiotics compared to 8 weeks post gavage. No other significant differences among taxa were noted (**Supplementary Table 3**).

## 4 Discussion

In this study we examined how mesalazine, prebiotics, or a combination of both affect fattening, glucose tolerance, and metabolic inflammation in ground squirrels and in outbred mice after microbiota transfer from ground squirrels. Prebiotics target the microbiota and have been shown to attenuate inflammation via effects on gut communities (Popov et al., 2013; Qin et al., 2023; Swiech et al., 2025). Mesalazine is a gut-specific NSAID that targets the initial inflammation at the gut level (Luck et al., 2015). Our treatments targeted the gut because the first steps in the development of obesity and metabolic inflammation are dysbiosis of the microbiota (reviewed in (Saad et al., 2016; Scheithauer et al., 2020)) and chronic low-grade inflammation in the gut (Rohm et al., 2021); reviewed in (Winer et al., 2016, 2017)). The metabolic inflammatory process generally begins when bacteria within the gut, mainly the cecum and colon, switch from a healthy, stable community with Bacteroidota (formerly Bacteroidetes) as the dominant phylum to a dysbiotic state made up mainly of Bacillota (formerly Firmicutes) (Ley et al., 2005). The dysbiotic gut leads to inflammation and stress in the adjacent intestinal mucosa which can be assessed by the tissue levels of inflammatory cytokines. We hypothesized that the combined treatment (mesalazine/prebiotics) would have the greatest effect on these parameters. Our data suggest, however, that the combined treatment does not have more impact than the individual treatments alone.

In terms of cytokines, the effects of treatments varied between species and gut compartment. Mesalazine increased TNF-α and IL-10 levels in ileum of squirrels, but not mice. This contradicts a previous study from our lab where mesalazine had a strong suppressive effect on TNF-α levels in squirrel ileum (Zur Tulod et al., 2023). Interestingly, the past and current studies both show a general trend for a decrease in these cytokines at later timepoints (Zur Tulod et al., 2023). The previous study also observed an increase in ileum IL-10 over time with mesalazine treatment, similar to what we observed here (Zur Tulod et al., 2023). The increased colon IL-10 with mesalazine that we measured here differs from the previous study, which found no change in this anti-inflammatory cytokine. These differences may be due to the differing lengths of treatment between these studies (10 weeks vs 8 weeks) and earlier initiation of the experiment relative to emergence from hibernation in the current study. In the DO mice, on the other hand, prebiotics with or without mesalazine had more effects overall on gut cytokines. The absence of effect of mesalazine is surprising because previous studies have shown a strong suppression of inflammation in the ileum and colon of mice treated with mesalazine (Luck et al., 2015). Unlike the Luck et al. study, however, the DO mice in our study received a GMT of 48-hr post-hibernation squirrel microbiota prior to treatment, which may have induced some inflammatory signaling. Luck et al. (2015) also used inbred mice on a HFD prior to and during treatment, while our mice were on a standard diet. We found lower levels of TNF-α in the colon of mesalazine-treated squirrels and mice after 8 weeks compared to 4 weeks of treatment, suggesting that the effects of mesalazine may accumulate over time. IL-10 levels in DO mice, but not squirrels, were highest in the groups treated with prebiotics with or without mesalazine and this effect was consistent throughout the study period. The concept of prebiotics as anti-inflammatory is not new. Supplementation with inulin decreases multiple proinflammatory cytokines, including TNF-α, in serum and ileum (Chambers et al., 2019; Shang, 2024). Prebiotic-treated squirrels also had higher levels of IL-10 in the colon at 4 weeks. This corresponds with previous research where anti-inflammatory cytokines increased with prebiotic treatment (Lindsay et al., 2006; Popov et al., 2013). Similarly, treatment with a combination of probiotics and prebiotics leads to lower pro-inflammatory cytokines (TNF-α, and IL-1α) (Furrie et al., 2005). Our results demonstrate different responses to these treatments between species in the ileum, although the trends are very similar in the colon. Overall, prebiotic treatment, more so than mesalazine, decreased inflammatory markers in the ileum of mice and the colon of both species. Also, the combined treatment did not have a greater effect than either treatment alone, and often correlated more with the prebiotics-only group than the mesalazine-only group. This suggests that prebiotic treatment had the strongest influence on cytokine levels.

Once gut inflammation is established, the gut barrier becomes leaky, which allows potentially harmful bacterial products into the bloodstream where they can interact with immune cells in other tissues (Gil-Cardoso et al., 2017). The most common product released by these bacteria is lipopolysaccharide (LPS), which binds to Toll-like receptors (TLR), specifically TLR4, and promotes low grade inflammation in the gut (Moya-Perez et al., 2015) and distant tissues, such as WAT (Jia et al., 2020; Xu et al., 2003). Studies of metabolic inflammation in other laboratory species sometimes examine blood levels of cytokines to examine systemic inflammation (Cani et al., 2009). Other common tests include insulin levels, homeostatic model assessment of insulin resistance (HOMA-IR), blood triglycerides, and adipokines, which were not examined in this study. We focused instead on body mass and tissue inflammation. Tissue-specific cytokine expression is an important aspect of metabolic inflammation in mice and humans (Gil-Cardoso et al., 2017; Hotamisligil et al., 1993; Khatib et al., 2014; Kunz et al., 2021). Furthermore, we have established previously that tissue inflammation occurs during fattening in the 13-lined ground squirrel (Sonsalla et al., 2021). Inflammation in WAT during obesity is generated by macrophages (Jia et al., 2020; Weisberg et al., 2003), mast cells (Lopez-Perez et al., 2021; Zelechowska et al., 2018), and T cells (de Jong et al., 2018; Nishimura et al., 2009; Strissel et al., 2010; Wu et al., 2019; Zeyda et al., 2011) that produce a number of proinflammatory cytokines (Feuerer et al., 2009; Strissel et al., 2010; Xu et al., 2003; Zelechowska et al., 2018). Treatment with mesalazine, prebiotics, or the combination of the two led to no changes in cytokine levels in the oWAT of ground squirrels or DO mice. This is interesting because past research from our lab and others has shown that mesalazine treatment decreases signs of inflammation in WAT of squirrels (Zur Tulod et al., 2023) and mice (Luck et al., 2015). This discrepancy could be due to differences in the lengths of the studies, dosage of mesalazine, or severity of intestinal inflammation. For ground squirrels, metabolic inflammation progresses the further they go into their active (fattening) season, leading to more adverse downstream effects (Sonsalla et al., 2021). Our previous mesalazine study in ground squirrels started about 2 months into the active season, when fattening was well underway (Zur Tulod et al., 2023). In the current study, we began treatment early in the active season (2 days post-emergence from hibernation), so metabolic inflammation had not yet begun in the squirrels. Thus, we may have prevented WAT inflammation rather than suppressing it. The mice also received a GMT of early active season microbiota from the same timepoint (2 days post-emergence squirrels). Unfortunately, we are unable to confirm that the microbiota transplant successfully engrafted in the mice, as we did not have a non-GMT mouse group for comparison. Although we were unable to statistically compare the community composition of the gavage material to that of the mouse cecum and colon microbial communities, visual inspection suggests the communities are quite similar. Thus, we speculate that the differences in responses to treatment between ground squirrels and mice are essentially species-specific differences.

Increasing fat deposits and increased pro-inflammatory cytokine levels throughout the body often lead to the development of insulin resistance (Hong et al., 2009; Hotamisligil et al., 1993; Kunz et al., 2021). Hibernators, such as ground squirrels, naturally become insulin resistant at the end of their active season to further increase adipose accumulation (Florant et al., 1985). In humans, insulin resistance (aka, glucose intolerance) occurs when a diet high in simple sugars or processed foods causes near-constant stimulation of the insulin receptors. This leads to downregulation of the receptors and subsequent resistance to insulin signaling. Initially, the β cells will release more insulin to compensate, but eventually will decrease insulin production, making blood glucose levels consistently high (Tabak et al., 2012). In this study, ground squirrels and DO mice were treated differently for GTT due to species differences in response to stressors (as described in Methods). Ground squirrels, but not mice, were anesthetized for the blood collections during the GTT due to the wild nature of the species. Anesthesia can significantly affect glucose tolerance (Diltoer and Camu, 1988; Laber-Laird et al., 1992; Vera et al., 2002). Despite this, we have successfully used anesthesia to monitor glucose tolerance in this species (Sonsalla et al., 2021; Zur Tulod et al., 2023). Because of the difference in handling between the species, we were careful not to make species-level comparisons in our analyses. In ground squirrels, we found no changes in glucose tolerance throughout the 8-week period examined. This is consistent with previous studies in this species which found glucose intolerance later in the season than was examined here (Sonsalla et al., 2021). Among the squirrel treatment groups, glucose tolerance fluctuated week to week with little consistency. In DO mice, glucose intolerance increased over the course of the study in the control, mesalazine, and mesalazine/prebiotic groups, but tended to stay steady in the prebiotics group. This trend appears to hold true for the ground squirrels as well, suggesting beneficial effects of prebiotics on insulin sensitivity, as has been reported in other studies (Bomhof et al., 2014; Neyrinck et al., 2012). The prebiotics used in this study, inulin and pectin, have been shown to improve glucose tolerance in adult humans (Chambers et al., 2019; Schwartz et al., 1988); therefore, this mixture of prebiotics may explain the maintenance of glucose tolerance in prebiotics-treated ground squirrels and mice.

Besides metabolic inflammation and insulin resistance, obesity and adiposity are most associated with the accumulation of fat deposits throughout the body. Increased pro-inflammatory cytokines lead to higher levels of serum triglycerides and low-density lipoproteins (LDL) in humans (Sherman et al., 1988). Metabolic inflammation promotes the deposition of these triglycerides in various tissues, including adipose (Park et al., 2020), liver (Salles et al., 2012; Yang et al., 1997), and skeletal muscle (Santanasto et al., 2015). During their active season ground squirrels rapidly accumulate mass to prepare for hibernation (Sonsalla et al., 2021; Zur Tulod et al., 2023). This accumulation of mass in the form of WAT allows hibernators to survive the long hibernation fast. We have previously found that mesalazine treatment increased body mass in ground squirrels during their fattening season (Zur Tulod et al., 2023). The current study, however, did not find any effect of treatment on body mass or adipose mass. The difference between the two studies could be due to timing. We began our study 48 hours after emergence from hibernation, whereas the previous study began mesalazine treatment 8 weeks later (Zur Tulod et al., 2023). Ground squirrel energy intake increased steadily in all groups over the study period in a similar way to what has been reported previously (Sonsalla et al., 2021). In DO mice, body mass also increased over the study, but energy intake remained constant. The steady increase in body mass is likely due to lean mass growth in relatively young mice. Interestingly, while adipose depot and liver mass did not change with any treatment in either species, the adipocyte size increased in the mesalazine/prebiotic-treated squirrels from the mid- to late timepoints. This may reflect a combination of the previously shown effects of mesalazine (Zur Tulod et al., 2023) and the increased energy harvest from the prebiotic-rich diet.

While the results of this study did not support our initial hypothesis that the mesalazine/prebiotic diet would reduce metabolic inflammation to the greatest extent, they did provide insights into further treatment for metabolic inflammation and obesity. Prebiotics had significant effects on tissue cytokines in both species, whether combined with mesalazine or administered alone. This suggests that inulin and pectin treatment supports a microbial community that promotes anti-inflammatory signaling. We found few effects of treatment on differential abundance of taxa in the mouse microbiotas. Where we saw effects, however, they were predominantly in mice treated with prebiotics, with or without mesalazine. Further study of the mechanisms behind this can help to design prebiotic treatments for patients with metabolic disease. Not all prebiotics have the same effects on metabolic inflammation. For example, low methylation pectins inhibit local and systemic inflammation, whereas high methylation pectins prevent intestinal inflammation (Popov et al., 2013). In the current study, we used high-methylated pectins, and using low-methylation pectins may affect inflammation differently. Extending the length of treatment may also positively affect the outcomes of the study. This study lasted 8 weeks; however, looking at later timepoints would show how the treatments affected inflammation long-term. While previous research found mesalazine to be effective in reducing metabolic inflammation, but not body mass gain, in ground squirrels later in the active season (Zur Tulod et al., 2023), it is still unknown how pectins may affect late active season animals. Overall, this study suggests that use of prebiotics, with or without anti-inflammatory drugs, can be a successful approach to treat or prevent metabolic inflammation, adiposity, and downstream co-morbidities.

## Data Availability

The raw data supporting the conclusions of this article will be made available by the authors, without undue reservation.
