# Supplementary material for: Anti-inflammatory drug and prebiotics alter metabolic inflammation in fattening ground squirrels and outbred mice
Source: Front Physiol. 2026 Jul 29;17:1802487. doi: 10.3389/fphys.2026.1802487 (PMC13461417; doi:10.3389/fphys.2026.1802487)
Supplement: Supplementary file 1 [file DataSheet1.pdf]

# *Supplementary Material*

## 1 Supplementary Figures and Tables

**Supplemental Table 1**. Ground squirrel and DO mice liver mass by sex. Numbers represent mean ± SEM.

| **Ground squirrels** | **Liver Mass (g)** | | | |
|---|---|---|---|---|
| ***Treatment*** | *Male (n)* | | *Female (n)* | |
| | 4 wk | 8 wk | 4 wk | 8 wk |
| Control | 7.243 ± 0.481 (3) | 6.807 ± 0.484 (3) | 5.768 ± 0.610 (4) | 5.760 ± 0.572 (5) |
| Mesalazine | 8.987 ± 1.401 (3) | 7.277 ± 1.767 (3) | 6.046 ± 1.047 (5) | 5.582 ± 0.666 (5) |
| Prebiotics | 6.360 ± 0.615 (3) | 7.110 ± 1.210 (3) | 5.214 ± 0.406 (5) | 5.594 ± 0.355 (5) |
| Mesalazine/Prebiotics | 7.363 ± 0.152 (3) | 7.555 ± 0.755 (4) | 5.246 ± 0.493 (5) | 7.547 ± 2.480 (3) |
| **DO Mice** | | | | |
| Control | 1.230 ± 0.015 (3) | 1.157 ± 0.499 (3) | 0.822 ± 0.042 (3) | 1.180 ± 0.125 (3) |
| Mesalazine | 1.085 ± 0.321 (4) | 1.290 ± 0.060 (4) | 0.965 ± 0.056 (4) | 1.107 ± 0.067 (3) |
| Prebiotics | 0.783 ± 0.323 (3) | 1.353 ± 0.092 (4) | 1.177 ± 0.076 (4) | 0.840 ± 0.239 (4) |
| Mesalazine/Prebiotics | 1.400 ± 0.139 (4) | 1.570 ± 0.046 (4) | 1.265 ± 0.156 (4) | 1.228 ± 0.118 (4) |

**Supplemental Table 2**. Ground squirrel and DO mice iaWAT mass by sex. Numbers represent mean ± SEM.

| **Ground squirrels** | **iaWAT Mass (g)** | | | |
|---|---|---|---|---|
| ***Treatment*** | *Male (n)* | | *Female (n)* | |
| | 4 wk | 8 wk | 4 wk | 8 wk |
| Control | 4.660 ± 1.406 (3) | 3.810 ± 0.661 (3) | 4.103 ± 0.245 (4) | 6.850 ± 0.961 (5) |
| Mesalazine | 6.217 ± 0.202 (3) | 4.080 ± 0.995 (3) | 6.138 ± 1.820 (5) | 6.422 ± 1.854 (5) |
| Prebiotics | 2.717 ± 0.686 (3) | 4.060 ± 1.219 (3) | 3.308 ± 0.301 (5) | 4.678 ± 0.920 (5) |
| Mesalazine/Prebiotics | 3.317 ± 0.280 (3) | 4.590 ± 0.944 (4) | 4.630 ± 0.978 (5) | 8.870 ± 3.456 (3) |
| **DO Mice** | | | | |
| Control | 0.357 ± 0.147 (3) | 0.900 ± 0.350 (3) | 0.370 ± 0.136 (3) | 0.457 ± 0.322 (3) |
| Mesalazine | 0.465 ± 0.106 (4) | 0.393 ± 0.098 (4) | 0.239 ± 0.053 (4) | 0.687 ± 0.375 (3) |
| Prebiotics | 0.340 ± 0.125 (3) | 0.578 ± 0.055 (4) | 0.368 ± 0.103 (4) | 0.308 ± 0.155 (4) |
| Mesalazine/Prebiotics | 0.448 ± 0.024 (4) | 1.255 ± 0.417 (4) | 0.113 ± 0.021 (4) | 0.293 ± 0.136 (4) |

Supplemental Table 3. **Differentially abundant taxa.** Differentially abundant taxa were determined using the ALDEx2 package in R.

| Comparison | Gut compartment | Taxon | diff.btw | Effect size |
|---|---|---|---|---|
| **Between Treatment** | | | | |
| Mesalazine vs Mesalazine + prebiotic (4week) | Cecum lumen | *Campylobacter* | -4.61 | -1.54 |
| Mesalazine vs Mesalazine + prebiotic (4week) | Cecum lumen | *Erysipelatoclostridum* | -4.54 | -1.21 |
| Mesalazine vs Mesalazine + prebiotic (4week) | Colon lumen | *Campylobacter* | -3.04 | -1.43 |
| Mesalazine vs Mesalazine + prebiotic (4week) | Colon lumen | *Anaerostipes* | -10.04 | -1.86 |
| Control vs Mesalazine (4week) | Colon lumen | Clostridia_UCG-014 | 8.59 | 2.01 |
| Mesalazine vs Prebiotic (4week) | Colon lumen | *Bacteroides* | 1.89 | 2.03 |
| Mesalazine vs Prebiotic (4week) | Colon lumen | *Campylobacter* | 3.47 | 1.55 |
| Mesalazine + prebiotic vs Prebiotic (8week) | Colon lumen | *Anaerotruncus* | -8.81 | -1.71 |
| **Within Treatment** | | | | |
| Prebiotic 4week vs 8week | Cecum lumen | Lachnospiraceae_UGC-001 | 5.56 | 1.41 |
| Prebiotic 4week vs 8week | Cecum lumen | uncultured Firmicutes | 3.48 | 1.46 |

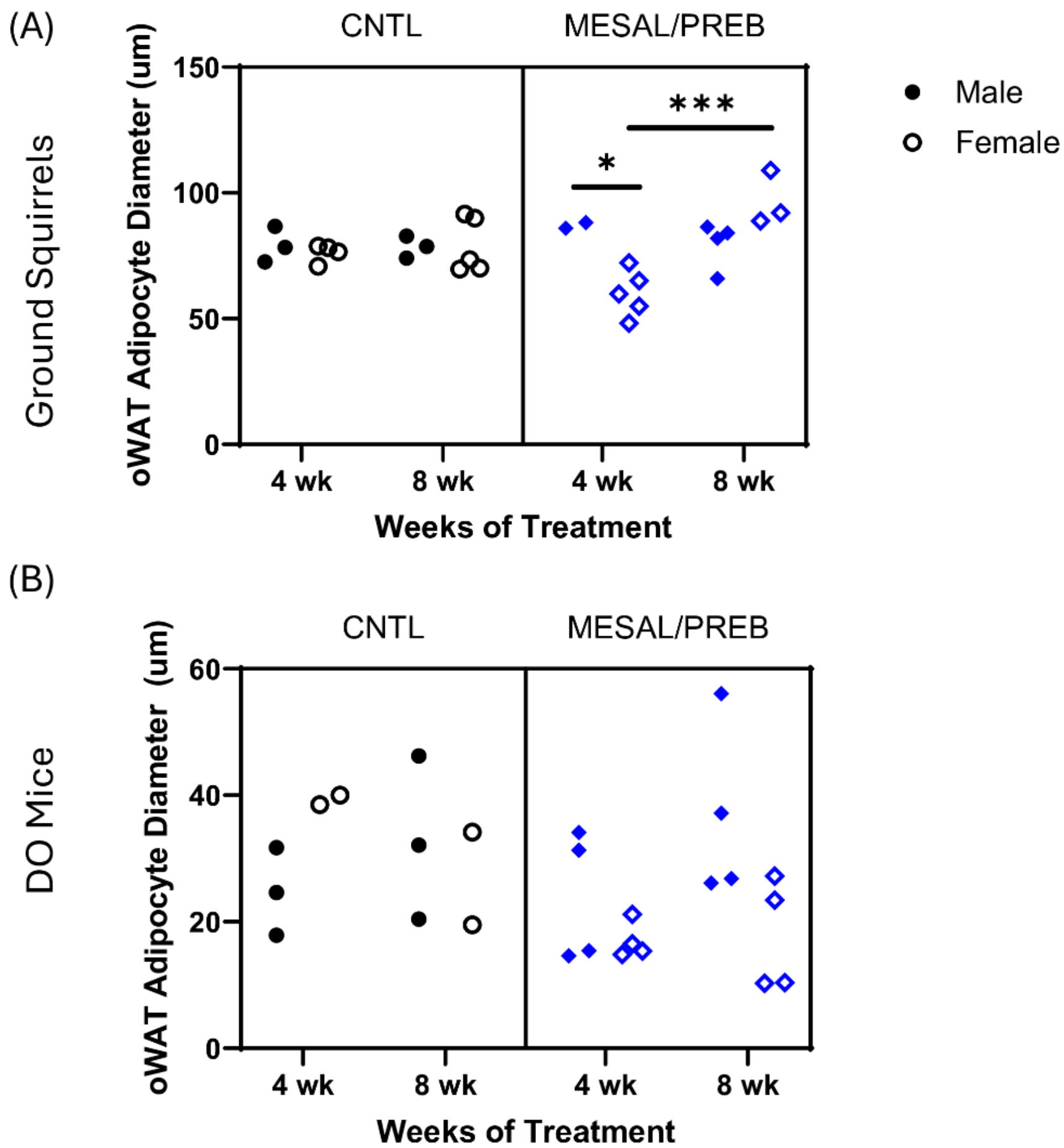


**Supplemental Figure 1**. Sex differences in oWAT adipocyte diameter in mesalazine/prebiotic treated ground squirrels (A, n = 2-5/group/timepoint) and DO mice (B, n = 2-4/group/timepoint). *, P ≤ 0.05, ***, P < 0.001.

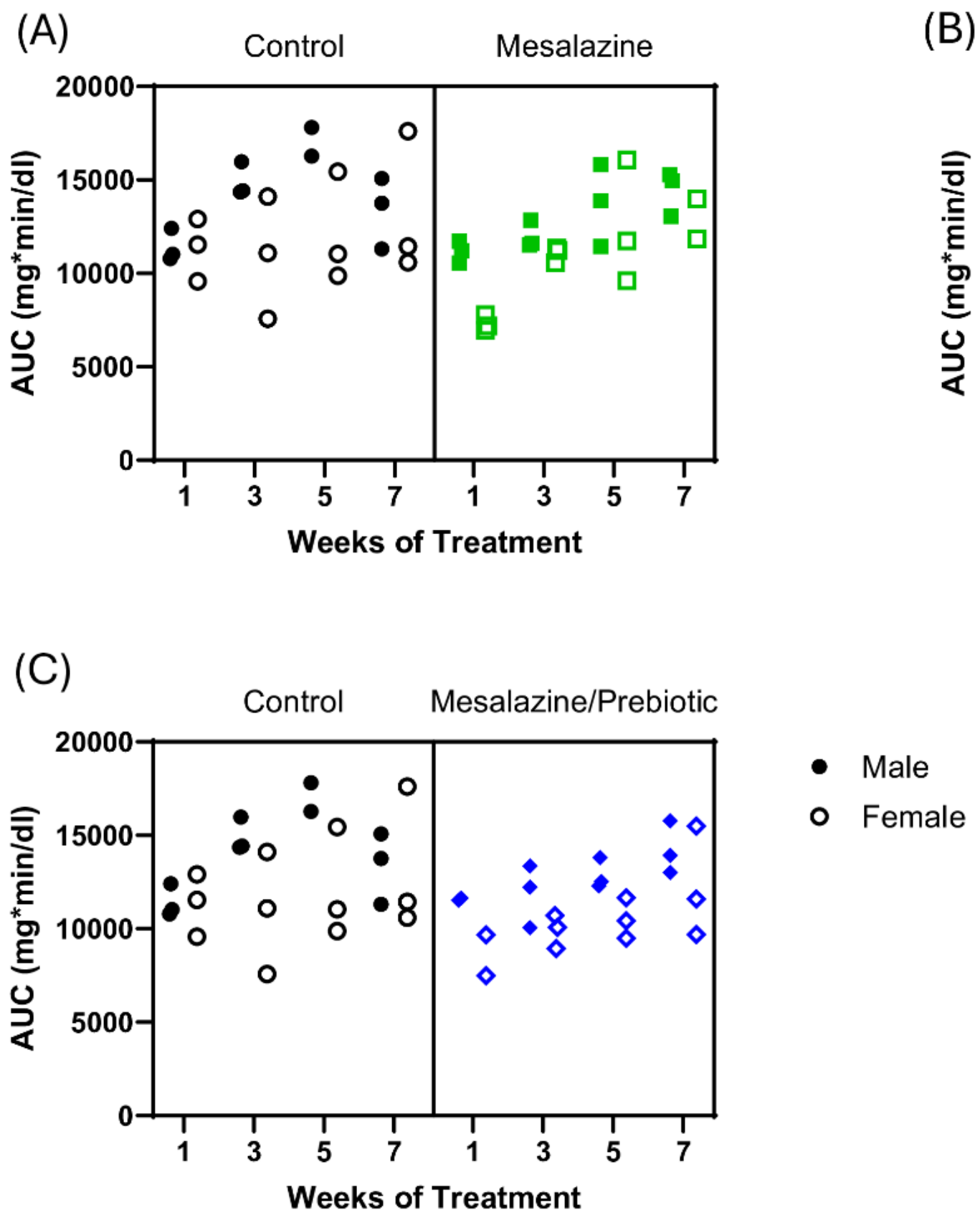


**Supplemental Figure 2**. Sex differences in glucose tolerance in DO mice under (A)mesalazine, (B) prebiotic, or (C) mesalazine/prebiotic treatment (n = 2-3/group/sex/timepoint).

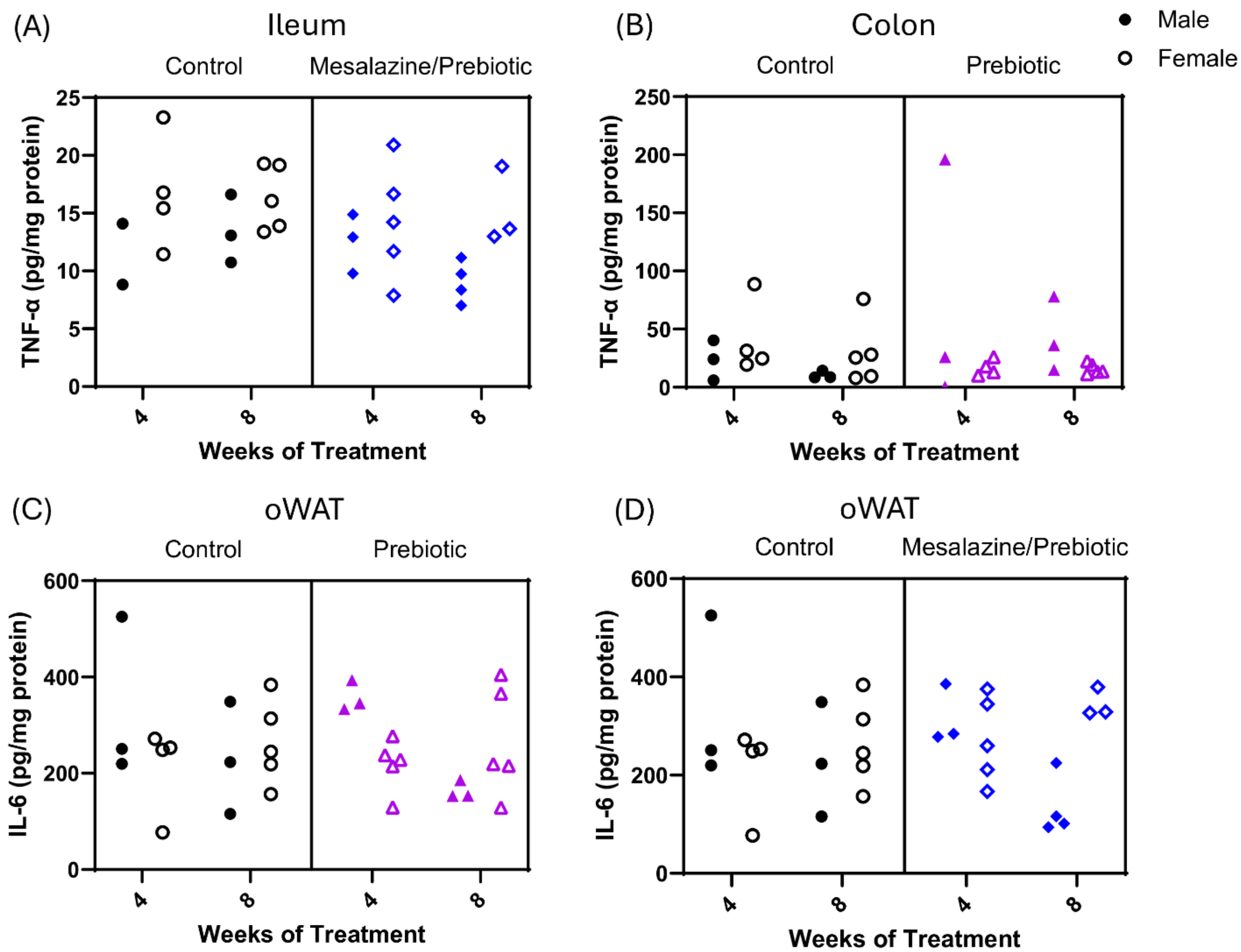


**Supplemental Figure 3**. Ground squirrel sex differences in (A) ileum TNF-α with mesalazine/prebiotic treatment ( n = 2-5/group/sex/timepoint), (B) colon TNF-α with prebiotic treatment (n = 3-5/group/sex/timepoint), (C) oWAT IL-6 with prebiotic treatment (n = 3-5/group/sex/timepoint), and (D) oWAT IL-6 with mesalazine/prebiotic treatment (n = 3-5/group/sex/timepoint).

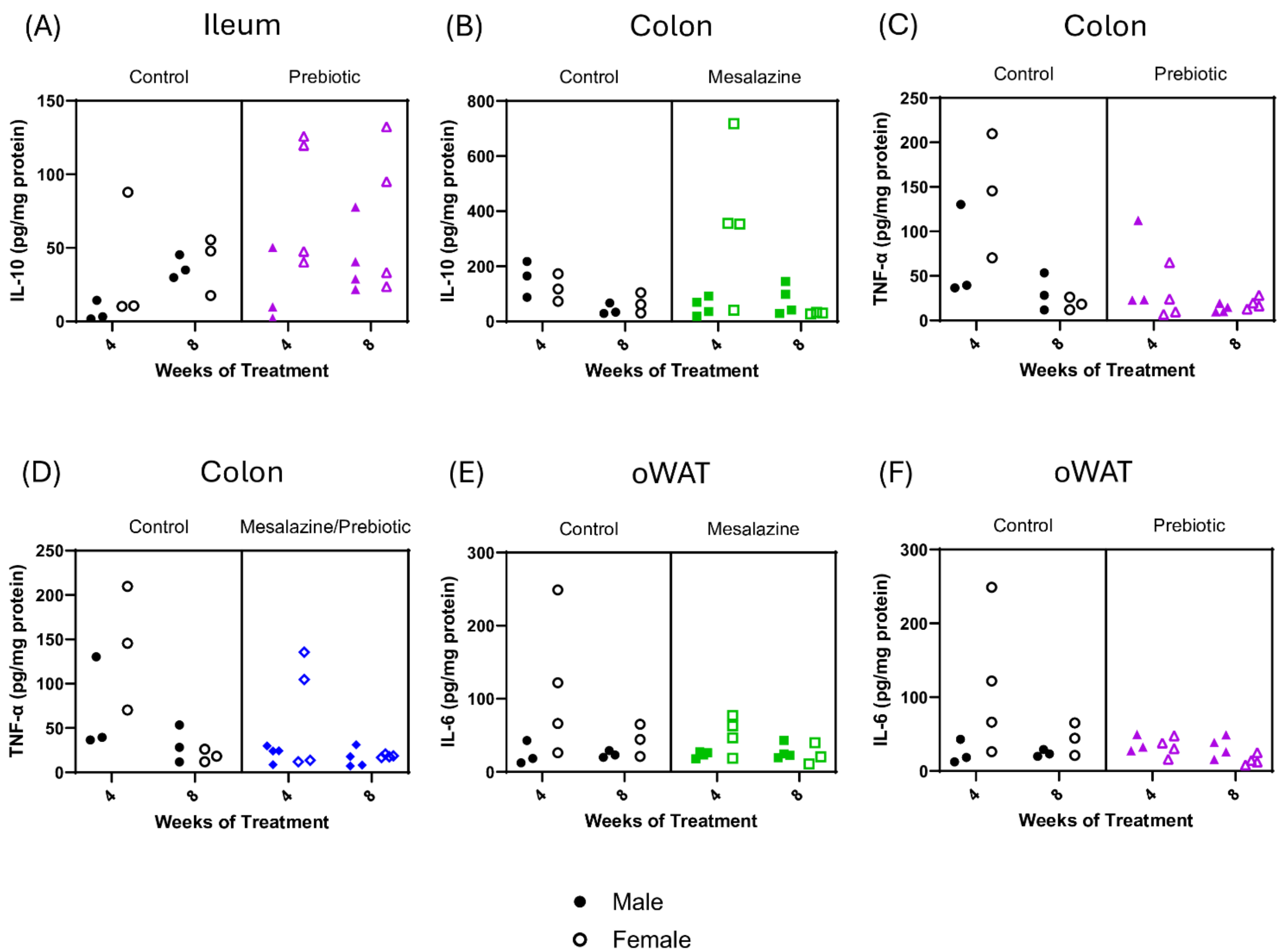


**Supplemental Figure 4**. DO mouse sex differences in (A) ileum IL-10 with prebiotic treatment (n = 3-4/group/sex/timepoint), (B) colon IL-10 with mesalazine treatment (n = 3-4/group/sex/timepoint), (C) colon TNF-α with prebiotic treatment (n = 3-4/group/sex/timepoint), (D) colon TNF-α with mesalazine/prebiotic treatment (n = 3-4/group/sex/timepoint), (E) oWAT IL-6 with mesalazine treatment (n = 3-4/group/sex/timepoint), and (F) oWAT IL-6 with prebiotic treatment (n = 3-4/group/sex/timepoint).

frontiers
